# Bromide of Ethyl

**Published:** 1880-07

**Authors:** 


Bromide of Ethyl.—Careful examination of current publications in regard to this anæsthetic has compelled us to modify the favorable opinion of its safety we expressed in a previous issue of this journal; and we now feel warranted in saying it should be resorted to with caution in the practice of surgery. The first unfortunate death from its administration that attracted our attention to its danger when administered as an anæsthetic, occurred in the practice of the world-wide gynæcologist, Dr. J. Marion Sims of New York. His frank and manly statements before the Academy of Medicine of that city, and their subsequent publication in the medical journals, of all the symptoms and circumstances connected with the unfortunate lady patient, went very far to convince us that it was *not* what we hoped, *a safe* anæsthetic at all times, at least; and though we thought it possible that the result of its administration in Dr. Sim's case might have been due to some latent, pre-existing morbid condition, or idiosyncrasy, that lay beyond the professional acumen of the distinguished surgeon that was called into activity by the ethyl; the result was calculated, nevertheless, to inspire caution in our mind when speaking or thinking about it as an anæsthetic, and we have been, consequently, deterred from using it where we desired to produce anæsthesia. But we we have more recent testimony regarding its danger, that will go far to lessen professional confidence in its use in surgery or obstetrics, if indeed it should not result in its entire abandonment as an anæsthetic agent.

Dr. R. J. Levis, who has done so much towards bringing it forward as a substitute for ether and chloroform, has had the misfortune to lose a patient recently in the Jefferson Medical College Hospital from its use. It seems to have been administered under his immediate supervision, to a patient upon whom he proposed operating for stone in the bladder, and the patient succumbed and died as the first incision was being made. This case would seem to settle the question of its danger as an anæsthetic, as Dr. Levis, with the single exception of Dr. Lawrence Turnbull, has had more experience with the drug than any other surgeon in this country, and probably in the world. We take this case of Dr. Levis' from the *Philadelphia Medical Times*, editorial of June 5th. Dr. Levis will probably feel himself called upon to furnish the particulars of the case, ere this shall reach our readers, and then they will be better able to decide how important a factor the ethyl was in causing the death of this patient.

